# The prion protein constitutively controls neuronal store-operated Ca^2+^ entry through Fyn kinase

**DOI:** 10.3389/fncel.2015.00416

**Published:** 2015-10-28

**Authors:** Agnese De Mario, Angela Castellani, Caterina Peggion, Maria Lina Massimino, Dmitry Lim, Andrew F. Hill, M. Catia Sorgato, Alessandro Bertoli

**Affiliations:** ^1^Department of Biomedical Science, University of PadovaPadova, Italy; ^2^CNR Neuroscience Institute, University of PadovaPadova, Italy; ^3^Department of Pharmaceutical Science, University of Piemonte OrientaleNovara, Italy; ^4^Department of Biochemistry and Genetics, La Trobe Institute for Molecular Science, La Trobe UniversityMelbourne, VIC, Australia

**Keywords:** prion protein, Ca^2+^, store-operated Ca^2+^ entry, Fyn kinase, cerebellar granule neurons, Alzheimer’s disease, Abeta oligomers, STIM1

## Abstract

The prion protein (PrP^C^) is a cell surface glycoprotein mainly expressed in neurons, whose misfolded isoforms generate the prion responsible for incurable neurodegenerative disorders. Whereas PrP^C^ involvement in prion propagation is well established, PrP^C^ physiological function is still enigmatic despite suggestions that it could act in cell signal transduction by modulating phosphorylation cascades and Ca^2+^ homeostasis. Because PrP^C^ binds neurotoxic protein aggregates with high-affinity, it has also been proposed that PrP^C^ acts as receptor for amyloid-β (Aβ) oligomers associated with Alzheimer’s disease (AD), and that PrP^C^-Aβ binding mediates AD-related synaptic dysfunctions following activation of the tyrosine kinase Fyn. Here, use of gene-encoded Ca^2+^ probes targeting different cell domains in primary cerebellar granule neurons (CGN) expressing, or not, PrP^C^, allowed us to investigate whether PrP^C^ regulates store-operated Ca^2+^ entry (SOCE) and the implication of Fyn in this control. Our findings show that PrP^C^ attenuates SOCE, and Ca^2+^ accumulation in the cytosol and mitochondria, by constitutively restraining Fyn activation and tyrosine phosphorylation of STIM1, a key molecular component of SOCE. This data establishes the existence of a PrP^C^-Fyn-SOCE triad in neurons. We also demonstrate that treating cerebellar granule and cortical neurons with soluble Aβ_(1–42)_ oligomers abrogates the control of PrP^C^ over Fyn and SOCE, suggesting a PrP^C^-dependent mechanizm for Aβ-induced neuronal Ca^2+^ dyshomeostasis.

## Introduction

The prion protein (PrP^C^) is an ubiquitous cell surface protein mostly expressed in the central nervous system, particularly at synapses (Um et al., 2012). Although it is now established that a conformational conversion of PrP^C^ generates the prion, the infectious particle causing fatal prion diseases in humans and animals (Prusiner, 1998), the physiological role played by PrP^C^ is still undefined. However, most of the proposed functions emphasize an involvement of PrP^C^ in surface receptor complexes transducing signals beneficial to the cell (Aguzzi et al., 2008; Linden et al., 2008). Accordingly, electrophysiological approaches and dye-based Ca^2+^ measurements in PrP-knockout (PrP-KO) or prion-infected cells have implicated PrP^C^ in the regulation of Ca^2+^ signaling (reviewed in Sorgato and Bertoli, 2009), which controls a wide range of physiological processes but also triggers cell death events if the fine Ca^2+^ tuning is compromised. Convincing evidence of the PrP^C^-Ca^2+^ liaison comes from the physical interaction of PrP^C^ with a subunit of the N-methyl-D-aspartate (NMDA)-sensitive glutamatergic receptors (NMDA-R), which reduces Ca^2+^ entry into hippocampal neurons and thus protects from glutamate excitotoxicity (Khosravani et al., 2008). PrP^C^ was also found to modulate Ca^2+^ influx in primary cerebellar granule neurons (CGN) after inducing store-operated Ca^2+^ entry (SOCE; Lazzari et al., 2011). SOCE occurs in response to depletion of Ca^2+^ from endoplasmic reticulum (ER), which causes the redistribution of ER luminal Ca^2+^ sensors (stromal interaction molecules, STIM1 and 2) into punctate clusters that recruit and activate the plasma membrane (PM) pore-forming proteins (Orai1-3) of store-operated channels (SOC; Soboloff et al., 2012). SOC mediate exclusively Ca^2+^ currents, which primarily serve to refill intracellular Ca^2+^ reservoirs but also to regulate different cell functions, including programmed cell death (Dubois et al., 2014), and gene expression (Lalonde et al., 2014) and excitation in neurons (Hartmann et al., 2014). It is thus reasonable to speculate that Ca^2+^ serves to mediate the different cell functions ascribed to PrP^C^ (Peggion et al., 2011).

It has also been claimed that the alleged receptor function of PrP^C^ relates to the modulation of phosphorylation cascades, in particular that governed by Fyn (Linden et al., 2008; Sorgato et al., 2009), a member of the Src Tyr-kinase family (SFK) highly expressed in neurons (Um and Strittmatter, 2013). Interestingly, because Tyr-phosphorylation regulates fundamental components of SOCE (Lopez et al., 2012), and the pharmacological inhibition, or deletion, of Tyr-kinases reduces SOCE-induced Ca^2+^ transients (Lee et al., 2006; Chung et al., 2007; Zuo et al., 2011) in various cell lines, one can hypothesize a tripartite connection between PrP^C^, Fyn and SOCE.

Recently, PrP^C^ was found to act as a cell surface binding partner for β-enriched protein aggregates, including amyloid-β (Aβ) oligomers (Laurén et al., 2009) related to Alzheimer’s disease (AD; Shankar et al., 2008), prions and other toxic oligomeric protein species (Resenberger et al., 2011). In this context, the PrP^C^-Fyn link has been proposed to mediate the effects of such neurotoxic entities. In particular, the PrP^C^-dependent activation of Fyn was identified as central to couple PrP^C^-Aβ complexes to NMDA-R redistribution, Ca^2+^ signaling alterations, tau hyper-phosphorylation, spine loss and death in neurons, and AD pathology in mice (Larson et al., 2012; Um et al., 2012).

To clarify PrP^C^ patho-physiology, in this study we sought to determine the capacity of PrP^C^ to govern local Ca^2+^ homeostasis in primary neuronal cultures after SOCE. We found that PrP^C^ regulates SOCE and SOCE-related Ca^2+^ movements in several cell domains by controling Fyn activation and the Tyr-phosphorylation of STIM1. We also observed that the control of PrP^C^ over Fyn and SOCE is subverted by soluble Aβ oligomers, suggesting that disruption of Ca^2+^ signaling mediates the PrP^C^-dependent effects of Aβ oligomers.

## Materials and Methods

### Animals

We used PrP-KO (line F10) mice and, as control, transgenic Tg46 (PrP-Tg) mice in which the expression of PrP^C^ at normal levels was rescued over the PrP-KO genotype (Mallucci et al., 2002; both strains kindly provided by the M.R.C. Prion Unit, London, UK). All aspects of animal care and experimentation were performed in compliance with European and Italian (D.L. 116/92) laws concerning the care and use of laboratory animals. The authors’ Institution has been accredited for the use of experimental mice by the Italian Ministry of Health, and by the Ethical Committee of the University of Padova.

### Primary Neuronal Cultures

CGN primary cultures were prepared (from 7 day-old mice killed by decapitation after anesthesia with methoxyflurane), cultured and transduced with lentiviral particles as previously described in Lazzari et al. (2011). Primary cultures of cortical neurons were obtained from cortices dissected from E18 mouse embryos. After carefully removing meningeal layers and blood vessels, the cortical tissue was gently minced and dissociated mechanically in B27 (2%)-added Hibernate E medium (Gibco). After centrifugation (180×g, 5 min), cells were suspended in Neurobasal medium (Gibco) supplemented with B27 (2%), horse serum (2%), glutamate (25 μM), L-glutamine (0.5 mM), and gentamycin [0.1 mg/ml (Gibco)], seeded on poly-L-lysine (100 μg/ml)-coated 13 mm-diameter glass coverslips at a density of 450,000 cell/cm^2^, and maintained at 37°C in a humidified incubator with 5% CO_2_. After 48 h, cells were infected with lentiviral particles and grown as previously described in Lazzari et al. (2011), except that cytosine-d-arabinofuranoside (10 μM) was added 72 h, and cells were used for experiments 144 h, after plating.

### Cell Cultures

HeLa and HEK-293T cells were grown in Dulbecco’s modified Eagle’s medium/High-Glucose (Euroclone), supplemented with 10% fetal bovine serum (Euroclone), 100 U/ml penicillin and 100 μg/ml streptomycin (Euroclone), and maintained at 37°C in a humidified incubator with 5% CO_2_. Twenty four hours before infection, HeLa cells were seeded onto 13 mm-diameter glass coverslips and allowed to grow to 70–80% confluence. For the production of lentiviral particles, HEK-293T cells were seeded onto 100 mm-diameter Petri dishes at ~40% confluence and transfected 24 h after plating, as described below.

### Construction of Lentiviral Vectors for Aequorins and Cell Transduction

To follow [Ca^2+^] fluctuations in specific neuronal compartments, we exploited a lentiviral expression system to transduce cells with chimeric constructs encoding the Ca^2+^-probe aequorin (AEQ) tagged with the influenza virus hemagglutinin (HA) epitope, and linked to sequences addressing the protein to the cytosolic domains proximal to the PM (AEQpm; Marsault et al., 1997; Lazzari et al., 2011), the bulk cytosol (AEQcyt; Brini et al., 1995), the ER lumen (AEQer; Montero et al., 1995; Lazzari et al., 2011) and the mitochondrial matrix (AEQmit; Rizzuto et al., 1992). Lentiviral vectors for AEQpm, AEQer and AEQmit were generated as described in Lim et al. (2008) and Lazzari et al. (2011), using an AEQ mutant with reduced Ca^2+^ affinity allowing [Ca^2+^] measurements up to hundreds of μM (Kendall et al., 1992). Conversely, a chimeric construct of wild-type (WT) AEQ fused to the monomeric red fluorescent protein (mRFP) was used to detect [Ca^2+^]_cyt_. To generate the AEQcyt lentiviral vector, two PCR reactions were performed. In the first one, the mRFP sequence was amplified without the stop codon using the pCDNA3-mRFP plasmid (Clontech) as template, and the following primers: XbaI-mRFP (forward: CGTCTAGAATGGCCTCCTCCGAGGAC) and mRFP-BglII (reverse: GAGGCGCCGGTGGAGTG-GAGATCTCG). In the second PCR, the HA-AEQ cassette was amplified using the pCDNA1-cytAEQ plasmid (Brini et al., 1995) as template, and the following primers: BglII-AEQ (forward: CGAGATCTCGAGCTCAAGCTTTATGA) and AEQ-SalI (reverse: GGTATCGATAAGCTTGATGTCGACGC). PCR products were digested with XbaI and BglII (for mRFP), or with BglII and SalI (for HA-AEQ), and the resulting fragments were assembled into the XbaI- and SalI-digested backbone of the lentiviral vector pRRLsin.PPTs.hCMV.GFP.pre in a three-step ligation reaction, yielding pLV-AEQcyt. Lentiviral particles were obtained as described previously (Lazzari et al., 2011). Briefly, HEK-293T cells were co-transfected with one of the transgene plasmids (pLV-AEQcyt, pLV-AEQmit, pLV-AEQpm or pLV-AEQer) together with the three packaging plasmids pMDLg/pRRE, pMD2.VSVG, pRSV-Rev (Naldini et al., 1996; Lazzari et al., 2011), using the calcium-phosphate method. After 10 h, the transfection medium was replaced with fresh culture medium, and after 72 h the culture medium was collected, and lentiviral particles were harvested by ultracentrifugation (50,000×g, 2 h), resuspended in phosphate-buffered saline (PBS; 140 mM NaCl, 2 mM KCl, 1.5 mM KH_2_PO_4_, 8 mM Na_2_HPO_4_, pH 7.4) and stored at −80°C. Viral titer was assessed by infecting HeLa cells by serial viral dilutions. Minimal dilutions adequate for obtaining 100% of infected cells were used to infect primary neurons and HeLa cells. All procedures for the production and use of lentiviral particles were performed in a biosafety level-2 environment. The expression and correct cellular localization of the Ca^2+^ probes were ascertained by immunocytochemical procedures, as described below and reported in Supplementary Figure 2.

### Measurements of Local Ca^2+^ Fluxes

The [Ca^2+^] range that can be reliably measured with the AEQ probes is defined by the type of AEQ (WT or mutated) and of the prosthetic group coelenterazine (Coe; normal (Coe-wt) or modified (Coe-n), Ottolini et al., 2014). The AEQ and Coe variants used in this work are listed in Supplementary Table 1.

For measuring [Ca^2+^]_pm_, [Ca^2+^]_cyt_ and [Ca^2+^]_mit_ transients, cells were firstly incubated (1 h, 37°C, 5% CO_2_) with Coe-wt (5 μM, Santa Cruz Biotechnology, cat. n. sc-205904) in a modified (Ca^2+^-free) Krebs-Ringer buffer (KRB, 125 mM NaCl, 1 mM Na_3_PO_4_, 1 mM MgSO_4_, 5.5 mM glucose, 5 mM KCl, 20 mM HEPES, pH 7.4) containing EGTA (100 μM) for simultaneously reconstituting functional AEQ and depleting intracellular Ca^2+^ stores. For the reconstitution of AEQer, cells were firstly incubated (10 min, 37°C) in the above Ca^2+^-free KRB containing EGTA (1 mM), and then incubated (50 min, 4°C) in Ca^2+^-free KRB supplemented with EGTA (500 μM), ionomycin (5 μM, Sigma) and Coe-n (5 μM, AnaSpec, cat. n. 82260).

Coverslips were then placed onto a recording chamber—equipped with a perfusion system—located into the luminometer. For the measurements of [Ca^2+^]_pm_, [Ca^2+^]_cyt_ and [Ca^2+^]_mit_, after a 1 min-perfusion step with EGTA (100 μM)-containing KRB, SOCE was elicited by perfusing cells with Ca^2+^ (1 mM)-containing KRB, as previously described in Lazzari et al. (2011). For measuring [Ca^2+^]_er_, cells were successively perfused with Ca^2+^-free KRB containing: EGTA (500 μM, 2 min); EGTA (1 mM) and bovine serum albumin [BSA 2% (w/v), 3 min]; EGTA (500 μM, 2 min); EGTA (100 μM, 1 min). CGN were finally stimulated for SOCE by perfusion with Ca^2+^ (1 mM)-containing KRB.

If needed, the inhibitors of voltage-gated Ca^2+^ channels (VGCC) [nifedipine (10 μM) and NiCl_2_ (50 μM or 1 mM); both from Sigma] were added to the perfusing buffer before (1 min) and during VGCC activation. Instead, the SFK inhibitors [PP2 (10 μM, Tocris) and saracatinib (5 μM, Santa Cruz Biotechnology)], and the negative control of PP2, PP3 (10 μM, Santa Cruz Biotechnology), were added during the reconstitution step, and kept in the perfusion buffer throughout the entire experiment. With the exception of NiCl_2_, all the above molecules were dissolved in dimethylsulfoxide [DMSO 0.1% (v/v)].

AEQ light emission was collected by means of an in-house built luminometer, equipped with a low-noise photo-multiplier coupled by an A/D board to a computer-assisted acquisition system, with a 1 Hz sampling rate (Ottolini et al., 2014). At the end of the recording, cells were permeabilized and exposed to saturating Ca^2+^ concentration by perfusion with KRB containing digitonin (50 μM, Sigma) and CaCl_2_ (10 mM). This allowed the calibration of the recorded light signal with respect to the total AEQ content, and its conversion into Ca^2+^ concentration, using the algorithm and the custom-made software previously described in Brini et al. (1995) and Montero et al. (1995).

### Immunocytochemistry

For immunocytochemical analyses, cells were washed in ice-cold PBS and fixed (20 min, RT) in 2% (w/v) paraformaldehyde (Sigma) in PBS. After extensive washing in PBS, cells were permeabilized with Triton X-100 [0.02% (w/v) in PBS, 1 h, RT], and then incubated (overnight, 4°C) with a mouse monoclonal antibody to the HA epitope [HA.11/clone16B12, Covance, cat. n. MMS-101P (1:250 in PBS)].

Cells were then washed in PBS, and incubated (1 h, 37°C) with AlexaFluor 488-conjugated anti-mouse antibody (1:500, Molecular Probes). Finally, coverslips were washed in PBS, mounted in Mowiol 40–88 [Sigma, 8% (w/v) in glycerol:PBS (1:3)] and observed with a confocal microscope system (Leica TCS SP5), which also allowed the acquisition and analysis of digital images.

### Preparation and Characterization of Aβ Oligomers

Chemically synthesized human Aβ_(1–42)_ peptides (Keck Laboratories) were dissolved (1 mg/ml), and incubated (1 h, RT), in 1,1,1,3,3,3-hexafluoro-2-propanol. The suspension was divided into solvent-free (by evaporation) aliquots (50 μg) and stored (−80°C). Just before use, peptides were dissolved in NaOH (20 mM, 50 μl), sonicated (15 min on ice), diluted with PBS (V_f_, 250 μl), and centrifuged (14,000×g, 5 min) to remove insoluble aggregates. After determining their concentration spectrophotometrically (λ, 214 nm), Aβ_(1–42)_ peptides were aged (1 h, 37°C) to form oligomers, and then administered to CGN or cortical neurons (1 h, 37°C) during the AEQ reconstitution step at a final concentration of 5 μM of monomer equivalents. Routinely, Aβ_(1–42)_ oligomerization was tested by Western blot (WB). For SDS-PAGE, Aβ_(1–42)_ samples (~300 ng), subjected, or not, to the aging protocol, were diluted in a buffer containing SDS [3% (w/v)], β-mercaptoethanol [1.5% (v/v)], glycerol [7.5% (w/v)], Coomassie blue G-250 [0.0125% (Serva)], Tris/HCl (37.5 mM, pH 7.0), and run in a urea (6M)-containing Tris-Tricine gel [16% (w/v) acrylamide-N,N′-methylenebisacrylamide (29:1)] (Schägger, 2006). Proteins were then electro-blotted onto polyvinylidene fluoride (PVDF) membranes [0.22 μm pore size (Biorad)], which were processed as described below, except for the blocking solution that contained non-fat dry milk [5% (w/v)] instead of BSA, and the primary mouse monoclonal antibody to Aβ_(1–42)_ (Covance, clone 6E10, cat. n. SIG 39320-200).

### Lysis of CGN and WB Analysis

To analyze the (activating auto-) Tyr-phosphorylation of SFK members (p-SFK) and of total neuronal proteins, CGN—treated, or not, with Aβ oligomers (see above)—were incubated (1 h, 37°C, 5% CO_2_) in KRB [supplemented with CaCl_2_ (1 mM), or EGTA (100 μM), to ensure protein analysis under the same conditions used to measure Ca^2+^ fluxes] in the absence, or in the presence, of the SFK inhibitors (PP2 and saracatinib), or PP3 (see above). CGN were lysed using an ice-cold buffer containing glycerol [10% (w/v)], SDS [2% (w/v)], Tris/HCl (62.5 mM, pH 6.8), urea (1.8 M), Na_3_VO_4_ (5 mM), and cocktails of protease and phosphatase inhibitors (Roche). Lysates, whose protein concentration was determined by a Lowry assay kit (Sigma, cat. n. TP0300), were then adjusted to an equal protein concentration using reducing (dithiothreitol, 50 mM) Laemmli sample buffer (Laemmli, 1970). After boiling (5 min), lysates were subjected to SDS–PAGE [10% (w/v) acrylamide-N,N′-methylenebisacrylamide (37.5:1)] and electro-blotted onto PVDF membranes [0.45 μm pore size (Millipore)], which were Coomassie-stained to verify equal loading and transfer (and for subsequent densitometric analyses, see below). PVDF membranes were then incubated (1 h, RT) with a blocking solution made of Tris-buffered saline added with Tween-20 (TBS-T) [0.1% (w/v)] and BSA [3% (w/v)], followed by addition of the appropriate primary antibody (4°C, over-night). In particular, to detect p-SFK, a rabbit polyclonal antibody (Cell Signaling Technology, cat. n. 2101) to p-Tyr416 was used. Instead, a mouse monoclonal antibody (clone P-Y20, Millipore, cat. n. 05-947) was used to detect the p-Tyr of total neuronal proteins. Simultaneously with these tests, the total amount of Fyn (in an equal CGN quantity) was determined using a specific rabbit polyclonal antibody (Cell Signaling Technology, cat. n. 4023). After three 10 min-washes (with TBS-T), membranes were incubated (1 h, RT) with a horseradish peroxidase-conjugated anti-rabbit or anti-mouse IgG secondary antibody (Santa Cruz Biotechnology, cat. n. sc-2004 and sc-2005, respectively). Immunoreactive bands were visualized and digitalized by means of a digital Kodak Image Station, using an enhanced chemiluminescence reagent kit (Millipore). For densitometric analyses, band intensities were normalized to the optical density of the corresponding lanes stained with Coomassie blue. To quantify the level of active Fyn, the normalized band intensity of p-SFK was divided by the normalized band intensity of Fyn. To electrophoretically separate different SFK members, equal amounts (10 μg) of CGN proteins were separated onto glycerol (35%)-supplemented SDS-PAGE gels [8% (w/v) acrylamide-N,N′-methylenebisacrylamide (37.5:1)]. After transferring proteins onto PVDF membranes [0.45 μm pore size (Millipore)], and the vertical cutting of membranes into strips, each strip was immunoprobed with a monoclonal antibody to either Src (Santa Cruz Biotechnology, cat. n. sc-5266), or Lck (Santa Cruz Biotechnology, cat. n. sc-433), while a polyclonal antibody was employed to label Lyn (Millipore, cat. n. 06-207), or Fyn, or p-SFK (indicated above). After incubation with the appropriate secondary antibody, PVDF membranes were accurately reconstructed before visualizing the immunoreactive signals. To detect PrP^C^, the mouse monoclonal antibody 8H4 was used (kindly provided by Prof. M.S. Sy, Case Western University, Cleveland, OH).

### Immunoprecipitation of STIM1

CGN—incubated (1 h, 37°C, 5% CO_2_) in KRB [supplemented with EGTA (100 μM) in order to deplete Ca^2+^ stores]— were lysed (30 min on ice) at 1 × 10^7^ cell/ml in a buffer containing Tris-HCl (20 mM, pH 7.4), NaCl (75 mM), EGTA (5 mM), Triton X-100 [1% (w/v)], sodium deoxycholate [1% (w/v)], Na_3_VO_4_, (5 mM) and cocktails of protease and phosphatase inhibitors. Cell lysates were sonicated and centrifuged (13,000×g, 15 min, 4°C) to remove insoluble materials, and the protein content of the collected supernatant was determined by a bicinchoninic acid-based assay kit (Thermo Scientific, cat. n. 23227). 300 μg of the supernatant proteins were then incubated (overnight, 4°C) with 1.5 μg of a monoclonal antibody to STIM1 (BD Bioscience, cat. n. 610954), followed by incubation with protein A-agarose (15 μl, SantaCruz Biotechnology, cat. n. sc-2001; 4 h, 4°C). The immunoadsorbant was centrifuged (4,000×g, 2 min, 4°C), washed twice with NaCl (100 mM), EDTA (2 mM), Na_3_VO_4_ (1 mM), and Tris-HCl (50 mM, pH 7.4), resuspended in reducing Laemmli buffer, boiled (3 min) and loaded onto Mini-protean TGX precast gels (4–15%, Biorad). Proteins were electro-blotted onto PVDF membranes [0.45 μm pore size (Millipore)], and immunodetected using, first, the antibody to p-Tyr (clone P-Y20, see above), and then, after stripping membranes with a commercial kit (Thermo Scientific, cat. n. 21059), a rabbit polyclonal antibody to STIM1 (Cell Signaling Technology, cat. n. 4916). Immunoreactive bands were visualized as described above, and for densitometric analyses the p-Tyr band intensity was normalized to that of STIM1.

#### Statistical Analysis

Values are reported as mean ± SEM. Data analysis was performed as described in Lazzari et al. (2011). Statistics were based on two-sample Student’s *t*-test, with a *p*-value < 0.05 being considered statistically significant.

## Results

### PrP^C^ Attenuates SOCE and SOCE-Induced Mitochondrial Ca^2+^ Uptake

Previously, comparison of primary CGN cultures obtained from WT and PrP-KO mice has demonstrated that the presence of PrP^C^ decreases SOCE-induced Ca^2+^ elevation in the cytosolic domains proximal to the PM (Lazzari et al., 2011). Here, we have extended the study to ascertain whether PrP^C^ also impinges on Ca^2+^ fluxes in other domains of primary CGN derived from PrP-KO, and as control, isogenic PrP-Tg mice (Supplementary Figure 1). Preliminarily, the correct sub-cellular localization of the differently targeted AEQ probes was confirmed by immunocytochemical approaches (Supplementary Figure 2).

**Figure 1:**
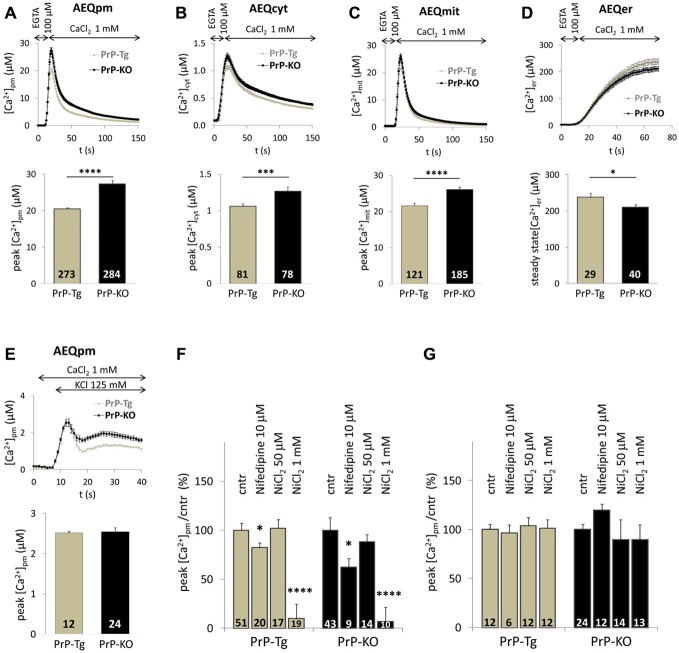
**PrP^C^ regulates Ca^2+^ movements in different cell domains of cerebellar granule neurons (CGN) after stimulation of SOCE. (A–C)** PrP-KO CGN (black) have higher Ca^2+^ fluxes than control PrP-Tg CGN (gray) in the cytosolic domains proximal to the plasma membrane (PM) **(A)**, the bulk cytosol **(B)** and the mitochondrial matrix **(C)**, as measured by the appropriate AEQ probe. Upper panels report the kinetics of local [Ca^2+^] transients, while the bar diagrams of the corresponding [Ca^2+^] peaks are shown in the lower panels. Here and after, the number of replicates (*n*) is indicated inside each bar diagram. **(D)** The steady-state [Ca^2+^] level in the endoplasmic reticulum (ER) lumen of PrP-KO CGN is significantly lower than in PrP-Tg CGN. In **(A–D)**, SOCE was activated by treating neurons with the Ca^2+^ chelator EGTA to deplete Ca^2+^ reservoirs. **(E)** Activation of VGCC by the 125 mM K^+^-depolarizing solution provokes similar [Ca^2+^]_pm_ transients and peaks in both CGN genotypes. **(F,G)** VGCC inhibitors (nifedipine and NiCl_2_) lower VGCC-induced [Ca^2+^]_pm_ peaks **(F)**, but leave unchanged those following SOCE **(G)**. *p < 0.05, ***p < 0.001, ****p < 10^−5^, Student’s *t*-test.

Figure 1 reports Ca^2+^ transients in PM microdomains (A), the bulk cytosol (B) and the mitochondrial matrix (C) following SOCE. While these data confirm that PrP-KO CGN are exposed to higher Ca^2+^ transients in PM microdomains than PrP-expressing neurons (Figure 1A), they also highlight that PrP-KO neurons display a significantly increased Ca^2+^ rise in the cytosol (Figure 1B) and in the mitochondrial matrix (Figure 1C). We argue that the higher Ca^2+^ levels observed in PrP-KO neurons are likely to be accounted for by both the more pronounced Ca^2+^ entry from the extracellular space (Figure 1A), and by the lower Ca^2+^-buffering capacity displayed by the ER (Figure 1D).

VGCC are highly abundant in neurons. To exclude their activation by local membrane depolarization following SOCE, we stimulated VGCC alone by perfusing neurons with either 25 mM or 125 mM K^+^ to mimic mild and strong depolarization, respectively. We found no detectable PM Ca^2+^ transients in CGN—expressing or not PrP^C^—under mild depolarizing conditions (data not shown). Conversely, the 125 mM K^+^ solution induced similar (<3 μM) PM Ca^2+^ peaks in both CGN types (Figure 1E) that, however, are much smaller than those observed upon SOCE. A second control envisaged treating neurons with inhibitors specific to L-type VGCC [nifedipine (10 μM)], or targeting all VGCC [NiCl_2_ (50 μM or 1 mM)]. We found that both inhibitors reduce Ca^2+^ entry stimulated by 125 mM K^+^ irrespective of the CGN genotype (~20% inhibition by nifedipine; ~95% inhibition by 1 mM NiCl_2_, Figure 1F). Instead, no significant diminution of PM Ca^2+^ peaks was observed in PrP-Tg and PrP-KO CGN when each inhibitor was added under the conditions employed to stimulate SOCE (Figure 1G). These results demonstrate that the contribution of VGCC to the observed Ca^2+^ transients is, if any, minimal.

### PrP^C^ Controls SOCE through Fyn

**Figure 2:**
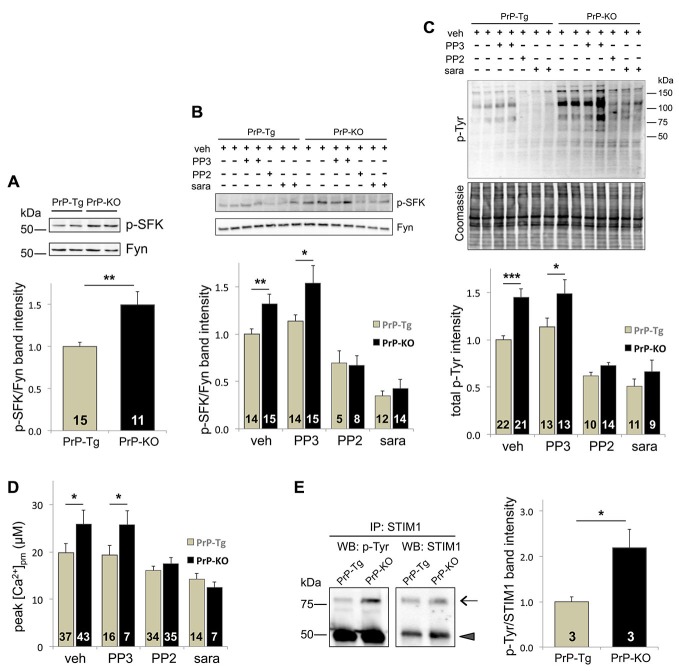
**The Tyr-kinase Fyn is the link between PrP^C^ and SOCE. (A)** PrP^C^ reduces the level of active Fyn, as evident from both the representative WB (upper panel) of PrP-Tg and PrP-KO CGN probed with an antibody to p-SFK or total Fyn (both run in duplicate), under SOCE-activating conditions (Ca^2+^-depleted stores), and the corresponding densitometric analysis of p-SFK immunosignals normalized to that of total Fyn (lower panel). Contrary to other neuronal SFK members (Supplementary Figure 4), the identical apparent mass of p-SFK and Fyn indicates that the Fyn band corresponds to the p-SFK band. Similar results were obtained under basal conditions, i.e., with Ca^2+^-filled stores (see Supplementary Figure 3). **(B,C)** Addition (+) of the SFK inhibitors saracatinib (sara, 5 μM) and PP2 (10 μM), but not of PP3 (10 μM), reduces the level of both p-SFK **(B)** and total Tyr-phosphorylated (p-Tyr) proteins **(C)** of CGN compared to the untreated (−) samples. **(B)** The upper panel reports a representative WB of the two neuronal genotypes treated with, or without, the SFK inhibitors, and immunostained as in **(A)**, while the lower panel shows the corresponding densitometric analysis of p-SFK normalized to the band intensity of total Fyn. Veh (vehicle) indicates the control experiment run in the presence of DMSO (0.1%). **(C)** The upper panel reports the WB of total p-Tyr proteins present in the two CGN genotypes treated as in **(B)**. In the corresponding densitometric analysis (lowest panel), the p-Tyr band intensity was normalized to that of the Coomassie blue-stained bands (middle panel). **(D)** Only saracatinib and PP2 decrease SOCE-induced [Ca^2+^]_pm_ peaks, and abrogate the difference observed in control (veh-treated) PrP-Tg and PrP-KO CGN. **(E)** STIM1 is more abundantly Tyr-phosphorylated in PrP-KO CGN than in PrP-Tg CGN under SOCE-stimulating conditions. This is evident from the representative WB (left panel) of immunoprecipitated (IP) STIM1 probed with an antibody to either p-Tyr or total STIM1 (arrow), and from the corresponding densitometric analysis (right panel) reported as the ratio between the p-Tyr band intensity and the STIM1 band intensity. The arrowhead in the left panels indicates the immunosignal of the mouse IgG used in the immunoprecipitation assay. On the left of the WB, MW standards are indicated. *p < 0.05, **p < 0.01, ***p < 0.001, Student’s *t*-test. The analysis of the statistical significance (*p*-value, Student’s *t*-test) of data for the comparison between different treatments within each PrP genotype **(B–D)** is reported in Supplementary Table 2. Other details are as in the legend to Figure 1.

Because of multiple reports linking PrP^C^ to Fyn (Mouillet-Richard et al., 2000; Santuccione et al., 2005; Pantera et al., 2009), we investigated whether Fyn signaling pathways critically acted in the observed regulation of PrP^C^ over SOCE. To inspect its activating auto-phosphorylation, we monitored the Tyr phosphorylation of SFK (p-SFK), demonstrating that p-SFK was at higher levels in PrP-KO CGN than in PrP-Tg neurons both with Ca^2+^-depleted stores (to trigger SOCE; Figure 2A), and under basal (i.e., with Ca^2+^-filled stores) conditions (Supplementary Figure 3). Albeit Fyn is particularly abundant in neurons (Um and Strittmatter, 2013), other SFK members are present (Lck, Lyn, Src, and Yes; Salter and Kalia, 2004). However, because among all tested SFK members Fyn is the only one co-migrating with the immunolabelled p-SFK band (Supplementary Figure 4), we can conclude that the observed p-SFK (Figure 2A and Supplementary Figure 3) corresponds to p-Fyn, and consequently, that PrP^C^ downregulates Fyn in CGN.

As expected, PP2 and saracatinib, two different selective inhibitors of SFK (but not PP3, the negative control of PP2), significantly diminish the level of p-Fyn (Figure 2B), and of all p-Tyr proteins (Figure 2C), in both CGN types. Importantly, these inhibitors also diminish SOCE-induced PM Ca^2+^ peaks, abrogating in this way the difference observed in untreated PrP-Tg and PrP-KO neurons (Figure 2D). The parallelism between SOCE and Fyn activations thus demonstrates that PrP^C^ regulates SOCE by controling the Fyn signaling pathways.

A key issue to unravel was identification of the molecular mechanizm linking SOCE to the PrP^C^-dependent control of Fyn. We focused on STIM1, one of the two ER Ca^2+^-sensor isoforms responsible for SOCE activation (Muik et al., 2012), not only because its Tyr-phosphorylation upregulates SOCE (Lopez et al., 2012), but also because the expression STIM1, which reaches its higher level in the cerebellum (Klejman et al., 2009), is predominant over STIM2 in mouse CGN (Lalonde et al., 2014). To mention also that the protocol of severe Ca^2+^ store depletion used here to fully activate SOCE is ideal to engage STIM1, whose Ca^2+^ affinity is much higher compared to STIM2 (Hoth and Niemeyer, 2013). Immunoprecipitation assays showed that, under the above-mentioned conditions, PrP-KO CGN display significantly more Tyr-phosphorylated STIM1 than PrP-Tg neurons (Figure 2E).

### Aβ_(1–42)_ Oligomers Impair PrP^C^-Dependent Control of SOCE through Fyn

**Figure 3:**
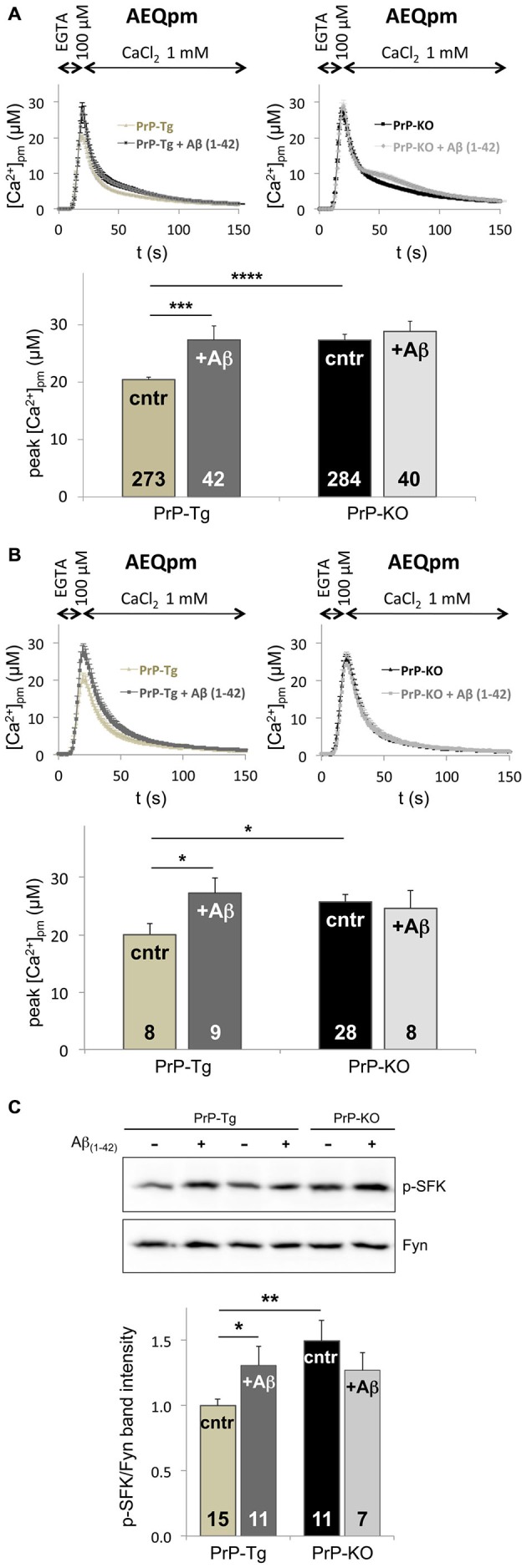
**Soluble Aβ_(1–42)_ oligomers increase SOCE and Fyn activation in a PrP^C^-dependent manner. (A,B)** Exposure to Aβ_(1–42)_ oligomers (at a concentration of 5 μM of monomer equivalents) increases the [Ca^2+^]_pm_ peaks only in PrP-expressing CGN **(A)** and cortical neurons **(B)** upon SOCE stimulation, thus abrogating the difference between the two genotypes observed in the absence of Aβ_(1–42)_ oligomers (cntr). **(C)** Under SOCE-triggering conditions, Aβ_(1–42)_ oligomers significantly increase the auto-phosphorylation of Fyn in PrP-Tg CGN, but not in PrP-KO CGN, nullifying the difference between the two genotypes observed in the absence of Aβ_(1–42)_ oligomers (cntr). This is apparent from both the representative WB of untreated (−), or Aβ_(1–42)_-treated (+), neurons (upper panel; probed with an antibody to p-SFK or total Fyn), and the densitometric analysis of the anti-p-SFK immunosignal normalized to that of total Fyn (lower panel). Similar results were obtained under basal conditions, i.e., with Ca^2+^-filled stores (see Supplementary Figure 6). *p < 0.05, **p < 0.01, ***p < 0.001, ****p < 10^−5^, Student’s *t*-test. Other details are as in the legend to Figures 1, 2.

Following the hypothesis that PrP^C^-Aβ interaction could be crucial for AD-related neuronal impairment (Laurén et al., 2009; Um and Strittmatter, 2013), we investigated whether soluble Aβ_(1–42)_ oligomers (Supplementary Figure 5) perturb the control of PrP^C^ over SOCE by monitoring PM Ca^2+^ transients. Compared to the untreated counterpart, we found that addition of soluble Aβ_(1–42)_ oligomers augments PM Ca^2+^ peaks of PrP-Tg CGN to the same value detected in untreated PrP-KO CGN (Figure 3A). Because no statistically significant effect was evident on Aβ-treated PrP-KO CGN, this result indicates that dysregulation of SOCE by oligomeric Aβ_(1–42)_ is strictly dependent on the presence of PrP^C^. Importantly, an identical PrP^C^-dependent alteration of SOCE was also observed in primary cortical neurons (Figure 3B), which are a primary target of Aβ oligomers in AD (Haass and Selkoe, 2007).

The capacity of Aβ_(1–42)_ oligomers to disturb SOCE only in PrP-Tg CGN was paralleled by the action on Fyn, because once again oligomeric Aβ_(1–42)_ increases the level of active Fyn in these neurons, both under SOCE-activating (Figure 3C) and basal (Supplementary Figure 6) conditions. This data indicates that soluble oligomeric Aβ_(1–42)_ increases SOCE by impairing the PrP^C^-dependent downregulation of Fyn.

## Discussion

By the novel comparison of local Ca^2+^ oscillations in isogenic primary CGN expressing, or not, PrP^C^, we report here that PrP^C^ restricts the accumulation of Ca^2+^ in the cytosol and mitochondria of neurons following SOCE. PrP^C^ accomplishes this task by limiting SOC-mediated Ca^2+^ entry and by increasing Ca^2+^ uptake by the ER, which likely depends on the capacity of PrP^C^ to upregulate the expression of the sarco/endoplasmic reticulum Ca^2+^-ATPase pump (Lazzari et al., 2011). We inferred that, within the used experimental paradigms, SOC are the only Ca^2+^ channels influenced by PrP^C^ because our control experiments show that VGCC do not significantly contribute to the observed Ca^2+^ fluxes (see also Park et al., 2010), nor that PrP^C^ changes the activity of VGCC in contrast to previous indications (Herms et al., 2000; Korte et al., 2003). Irrespective of this aspect, our results also highlight the considerable capacity of CGN mitochondria to buffer SOC-mediated Ca^2+^ influx, undisclosed in neurons thus far, in accord to the connection between the mitochondrial Ca^2+^ uniporter and SOCE observed in mast cells (Samanta et al., 2014).

Our data emphasize the protective function of PrP^C^ towards perilous local Ca^2+^ overloads (Khosravani et al., 2008) that may undermine neuronal functions and plasticity, especially in neurodegenerative disorders (Berridge, 2014). Consistent with this view, while normal SOCE was found to maintain the structure of mushroom spines (pivotal to learning and memory; Sun et al., 2014), excessive SOCE was implicated in hypoxia-induced neuronal death (Berna-Erro et al., 2009). Likewise, the capacity of PrP^C^ to shape local Ca^2+^ signals may shed light into neurodegenerative pathways such as those occurring during prion infection, where changes in the expression level and processing of PrP^C^ (Mays et al., 2014) may contribute to Ca^2+^-induced neuronal damages.

The PrP^C^-dependent downregulation of SOCE was also observed in primary cortical neurons, suggesting that PrP^C^ controls SOCE and intracellular Ca^2+^ transients in different neuronal cell types. In this context, it is to be mentioned that, although SOCE is a major pathway for Ca^2+^ entry in non-excitable cells (Parekh and Putney, 2005), the importance of SOCE is increasingly recognized also in excitable cells, including neurons (Moccia et al., 2015), in which its role is just beginning to be fully deciphered (Majewski and Kuznicki, 2015). In particular, a recent report suggests that SOCE could also regulate gene expression through the transcription factor Sp4 (Lalonde et al., 2014), which is known to contribute to complex neuronal processes including learning and memory (Zhou et al., 2010). Our results thus suggest that the modulation of SOCE could be one of the means connecting PrP^C^ to the different neuronal functions attributed to the protein.

Mechanistically, we identified Fyn as a molecular intermediate enabling PrP^C^ to control SOCE in light of the observed close correlation between Fyn and SOCE: both are upregulated in the absence of PrP^C^, while both are downregulated by selective SFK inhibitors (PP2 and saracatinib). In addition to the mandatory variation in ER Ca^2+^ levels, SOCE is regulated by different mechanizms that include the glutathionylation and phosphorylation of STIM proteins (Hawkins et al., 2010; Pozo-Guisado and Martin-Romero, 2013). In particular, the Tyr-phosphorylation of STIM1 by SFK members increases SOCE (Lopez et al., 2012). We report here that—under SOCE-triggering conditions—STIM1 is more Tyr-phosphorylated in PrP-KO than in PrP-expressing neurons, a result fully consistent with the higher activation of SOCE and Fyn observed in PrP-KO neurons. Although the precise site(s) of Tyr-phosphorylation on STIM1 is(are) unknown, Tyr361 in the cytosolic domain of the protein appears the most likely target of SFK members. This site was found to be phosphorylated by mass spectrometry studies (http://www.phosphosite.org/siteAction.do?id=25755096), is highly conserved among different mammalian species, and is also present in STIM2. Further studies will clarify if Tyr361 is the actual phosphorylation site by SFK and if this is a mechanizm to functionally regulate both STIM isoforms.

Multiple indications have implicated Fyn as a downstream effector of PrP^C^ in the regulation of key cell processes, ranging from embryogenesis and neuritogenesis to, at large, neuroprotective signaling (Aguzzi et al., 2008; Linden et al., 2008). However, while most observations were obtained upon stimulation of cells [e.g., by antibody-mediated clustering of PrP^C^ (Mouillet-Richard et al., 2000; Pantera et al., 2009), or upon binding of PrP^C^ to NCAM (Santuccione et al., 2005)], for the first time to our knowledge we have shown that PrP^C^ depresses Fyn activity under basal conditions. This result opens the possibility that PrP^C^ is constitutively implicated in such a crucial aspect of cell physiology, and that dysregulation of this function may be particularly relevant for those disease-related species, like Aβ oligomers and prions, which have been proposed to exploit PrP^C^ as surface binding partner for the downstream transduction of their toxicity (Barton and Caughey, 2011; Wang et al., 2013; Hirsch et al., 2014).

In AD, both Ca^2+^ dyshomeostasis (Green and LaFerla, 2008; Demuro et al., 2010) and aberrant Fyn signaling (Lambert et al., 1998; Roberson et al., 2011) were indicated to mediate the deleterious effects of oligomeric Aβ. Accordingly, it was shown that Fyn, which is part of a super-molecular complex with PrP^C^ and the metabotropic glutamate receptor 5 (mGluR5, serving to connect Fyn and PrP^C^ on the opposite sides of the PM), is activated after Aβ docking to PrP^C^ (Larson et al., 2012; Um et al., 2012, 2013). We found that exposure to soluble Aβ_(1–42)_ oligomers disrupts the PrP^C^-Fyn-SOCE triangle because such a treatment increases activation of both Fyn and SOCE in PrP-expressing, but not in PrP-KO, CGN. Importantly, the PrP^C^-dependent effect of Aβ oligomers on SOCE was also observed in cortical neurons that, together with hippocampal neurons, are the preferential target of Aβ toxicity (Haass and Selkoe, 2007).

Considering the influence of PrP^C^ on Fyn, our findings are reminiscent of Fyn activation by PrP^C^ cross-linking (Mouillet-Richard et al., 2000) or PrP^C^-NCAM clustering (Santuccione et al., 2005). However, they disclose a different underlying mechanism, whereby the interaction of PrP^C^ with extracellular ligands releases the basal block of PrP^C^ on Fyn rather than the ligand-PrP^C^ complex directly promoting Fyn activation. Hence, one initial step of oligomeric Aβ_(1–42)_ toxicity could involve PrP^C^ displacement from the role of sentinel against neuronal Ca^2+^ overload. Additional studies are needed to clarify whether this effect is consequent to a structural modification of PrP^C^, or a dislodgment from natural interacting partners, or a modification of the membrane lipid architecture surrounding the protein. Likewise, also the link between Fyn and PrP^C^ in CGN needs to be elucidated. We exclude the involvement of mGluR5 (Um et al., 2013), given that the neurons utilized here do not harbor detectable amounts of mGluR5 nor respond to archetypal mGluR5 agonists (unpublished observations).

In conclusion, we report here that exposure of neurons to oligomeric Aβ_(1–42)_ results in increased activation of Fyn and SOCE. However, given that PrP-KO mice show no gross phenotype, nor overt signs of neurodegeneration, the alterations observed in this work cannot be sufficient to account for AD pathology. Nonetheless, they could act as necessary events that, combined with other (PrP^C^-dependent and/or PrP^C^-independent) insults by Aβ oligomers, eventually contribute to AD-related neuronal damage. Further studies will be necessary to demonstrate whether Aβ oligomers perturb local Ca^2+^ fluxes different from SOCE, in particular those arising from NMDA-R that have already been functionally associated to PrP^C^ (Khosravani et al., 2008).

## Author Contributions

MCS, AFH and AB designed research; ADM, AC, CP, MLM and DL performed research (ADM and AC contributed equally); ADM, CP and AB analyzed data; AB, AFH and MCS wrote the article.

## Funding

This work was supported by the Italian Ministry of University and Research (PRIN/2010 to DL), the University of Padova (PRAT CPDA121988/12 to MCS), and the Australian Academy of Sciences (to AFH).

## Conflict of Interest Statement

The authors declare that the research was conducted in the absence of any commercial or financial relationships that could be construed as a potential conflict of interest.
