# Supplementary material for: The prion protein constitutively controls neuronal store-operated Ca2+ entry through Fyn kinase
Source: Front Cell Neurosci. 2015 Oct 28;9:416. doi: 10.3389/fncel.2015.00416 (PMC4623396; doi:10.3389/fncel.2015.00416)
Supplement: Supplementary file 1 [file Presentation_1.pdf]

Supplementary material

The prion protein constitutively controls neuronal store-operated Ca^{2+} entry through Fyn kinase

Agnese De Mario[1], Angela Castellani[1], Caterina Peggion[1], Maria Lina Massimino[2], Dmitry Lim[3], Andrew F. Hill[4], M. Catia Sorgato[1,2*], and Alessandro Bertoli[1*]

[1]Department of Biomedical Science, University of Padova, 35131 Padova, Italy

[2]CNR Institute of Neuroscience, University of Padova, 35131 Padova, Italy

[3]Department of Pharmaceutical Science, University of Piemonte Orientale, 28100 Novara, Italy

[4]Department of Biochemistry and Genetics, La Trobe Institute for Molecular Science, La Trobe University, VIC 3086 Melbourne, Australia

[*]**Correspondence:**
Alessandro Bertoli or M. Catia Sorgato
Dept. of Biomedical Science
University of Padova
Via U. Bassi 58/B, 35131 Padova, Italy
alessandro.bertoli@unipd.it
catia.sorgato@unipd.it

Supplementary Figures

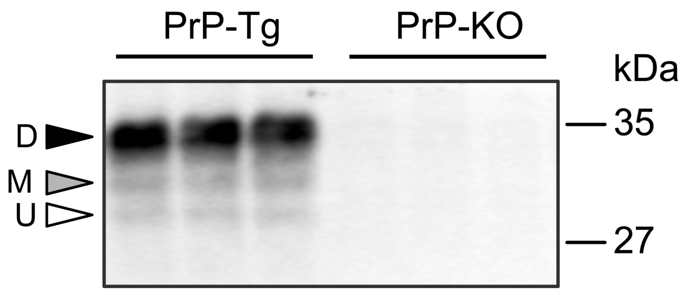

Supplementary Figure 1. WB of CGN probed with an antibody to PrP^C confirms that PrP^C is expressed in PrP-Tg CGN, but not in the PrP-KO counterpart (both genotypes run in triplicate). Shown data are representative of at least ten independent experiments that yielded comparable results. Arrowheads on the left indicate the three PrP^C glycoforms (di-, D; mono-, M; un-glycosylated, U), while MW standards are indicated on the right.

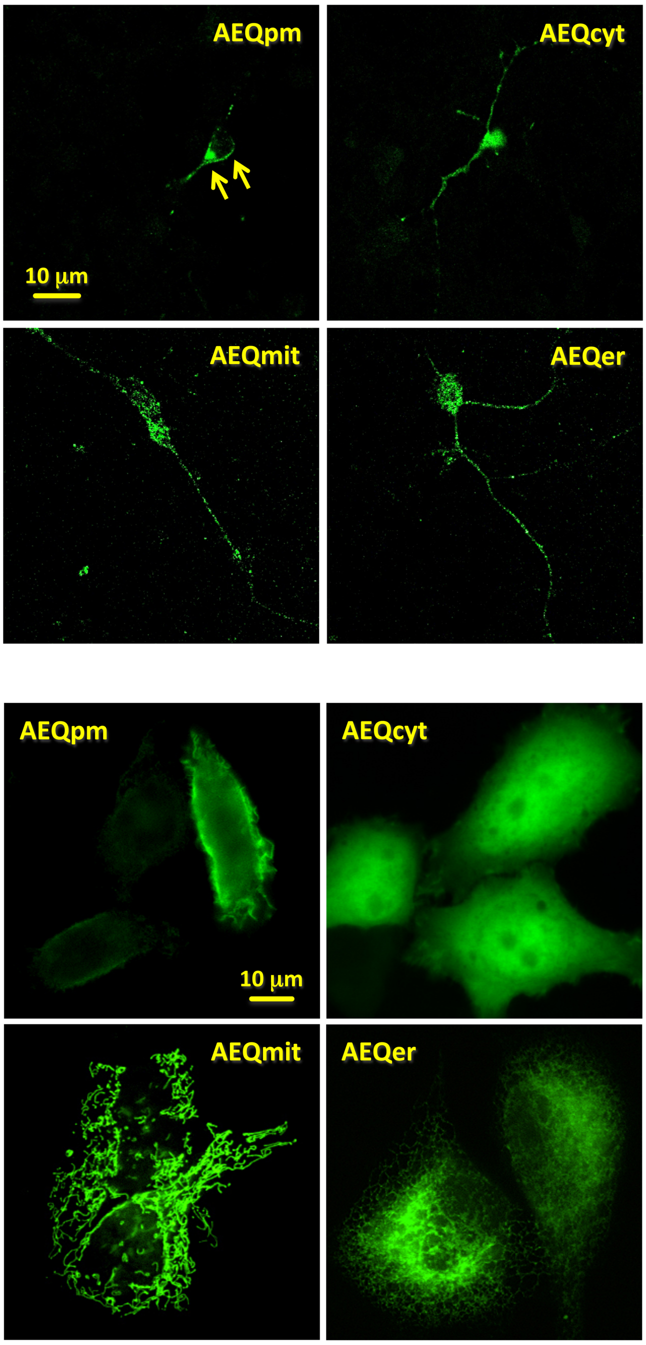

Supplementary Figure 2. The immunocytochemical analysis of primary CGN (upper panels) and HeLa cells (lower panels) – using an antibody to the AEQ-tagging HA epitope – demonstrate the correct sub-cellular targeting of all used Ca^{2+}-probes. Both CGN and HeLa cells were infected with the same lentiviral vectors encoding the different AEQ isoforms. The localization of AEQpm (note the staining of the cell soma rim highlighted by the arrows) and AEQcyt in CGN is readily appreciable. The small dimensions of these cells, however, do not allow to easily distinguishing AEQmit and AEQer distribution. The sub-cellular localization of the different AEQ probes is better discernible in the much larger HeLa cells.

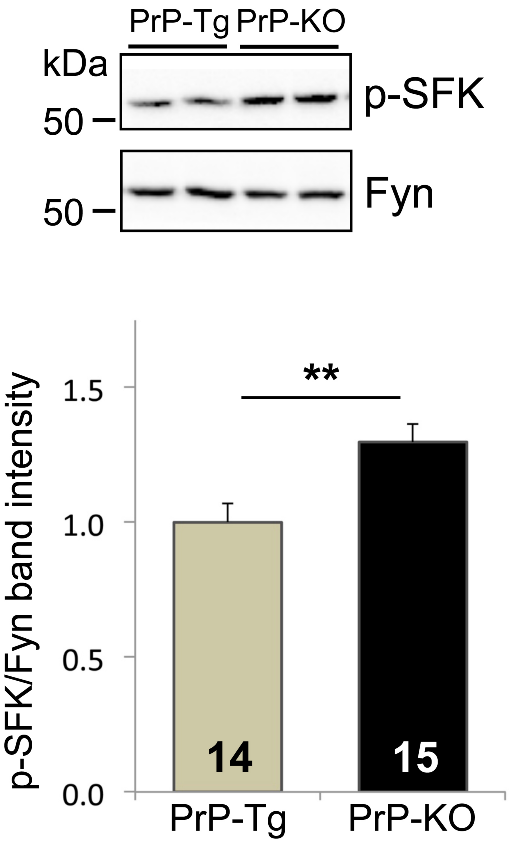

Supplementary Figure 3. PrP^C reduces the level of active Fyn under basal conditions (i.e., in the presence of 1 mM Ca^{2+}). This is shown in the representative WB (upper panel) of CGN immunostained with an antibody to p-SFK or total Fyn (both run in duplicate), and in the corresponding densitometric analysis of the anti-p-SFK immunosignal normalized to that of total Fyn (lower panel). On the left of the WB, MW standards are indicated. Here and after, the number of replicates is indicated in each bar diagram. ** $p<0.005$, Student's t-test.

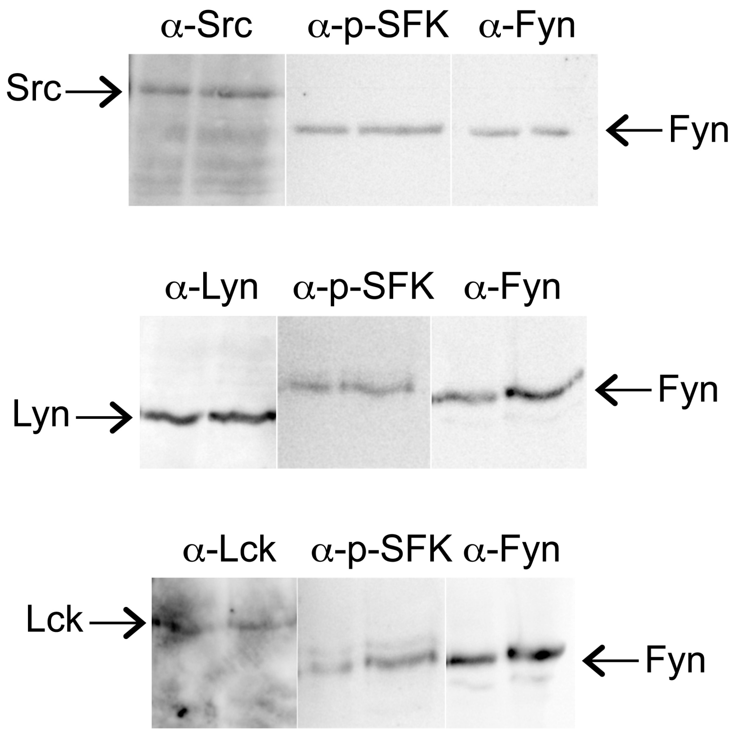

Supplementary Figure 4. Fyn, but not other Src family kinases (Src, Lyn, Lck), co-migrates with p-SFK in SDS-PAGE. An equal protein amount of PrP-Tg CGN was loaded (in 6 replicates) into three SDS-PAGE gels (run in parallel) and electro-blotted onto PVDF membranes. Each membrane was then vertically cut into three identical parts, and each strip, always probed with an antibody (α) to p-SFK, was alternatively probed with an antibody to total SFK members: Src and Fyn (upper panel); Lyn and Fyn (middle panel); Lck and Fyn (lower panel). Following the accurate reconstitution of the original membranes before visualization of the immunoreactive bands, shown data demonstrate that Fyn is the only one member migrating with the same apparent mass of p-SFK. Considering that also Yes, another SFK member possibly present in CGN (which was not tested), has a theoretical mass quite different from that of Fyn, this experiment indicates that the p-SFK immunosignals reported in Figs. 2A, 2B and 3C correspond to p-Fyn. Shown data are representative of three independent experiments that yielded comparable results.

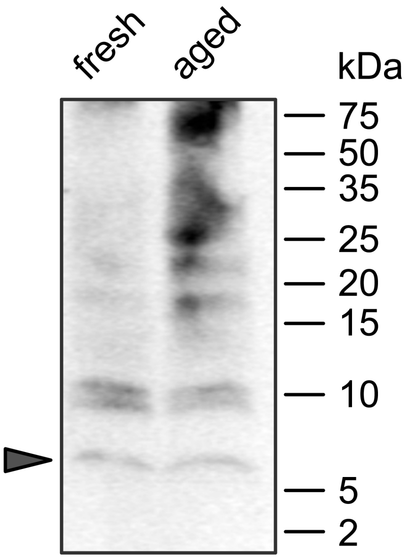

Supplementary Figure 5. Representative WB showing the oligomerization process occurring during the "aging" of A$\beta_{(1-42)}$. Equal amounts (300 ng) of fresh or "aged" A$\beta_{(1-42)}$ samples were separated by Tris-Tricine gel electrophoresis, blotted onto a PVDF membrane and stained with an anti- A$\beta_{(1-42)}$ antibody. The process of aging (1h, 37 °C) clearly increases high mass oligomeric forms with respect to the monomeric form (arrowhead on the left). Each A$\beta_{(1-42)}$ preparation was subjected to this type of analysis that provided similar results. MW standards are reported on the right.

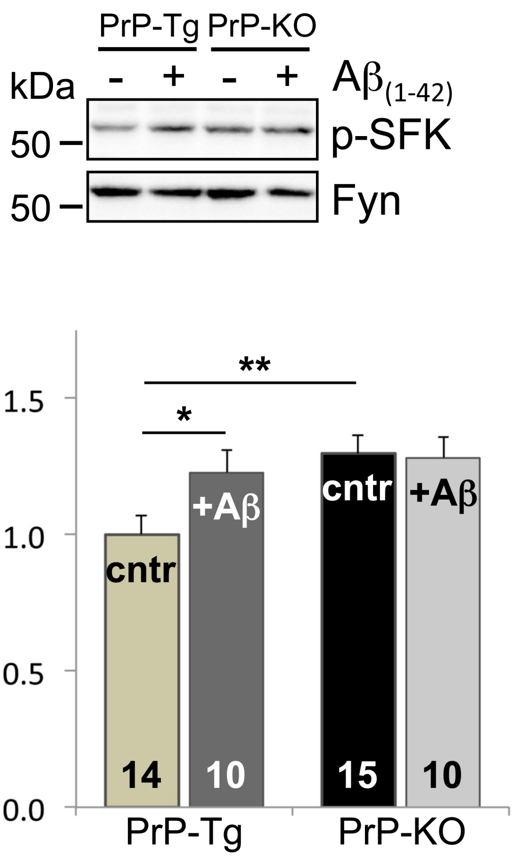

Supplementary Figure 6. Treatment with $A\beta_{(1\text{-}42)}$ oligomers (at a concentration of 5 μM of monomer equivalents) significantly increases Fyn auto-phosphorylation in PrP-Tg, but not in PrP-KO, CGN under basal conditions (1 mM Ca^{2+}), and abrogates the difference between the two genotypes observed in the absence of $A\beta_{(1\text{-}42)}$ oligomers. This is shown by the representative WB of p-SFK and total Fyn of control (-), or $A\beta_{(1\text{-}42)}$-treated (+), PrP-Tg and PrP-KO CGN (upper panel), and the densitometric analysis of the corresponding anti-p-SFK immunosignals normalized to that of total Fyn (lower panel). MW standards are reported on the left of the WB. * $p<0.05$, ** $p<0.005$, Student's t-test.

Supplementary Tables

Supplementary Table 1. AEQ probes for [Ca^{2+}] measurements in different sub-cellular compartments. Depending on the targeted compartment, different aequorins (AEQ) [WT or mutated (mut)], and coelenterazine [normal (Coe-wt) or modified (Coe-n)], were used, allowing the reliable measurement of different [Ca^{2+}] ranges.

Probe name	AEQ type	Coe type	pLV name	Compartment	[Ca^{2+}] range
AEQpm	AEQ-mut	Coe-wt	pLV-AEQpm	sub-PM domains	2-100 µM
AEQcyt	AEQ-WT	Coe-wt	pLV-AEQcyt	cytosol	0.5-10 µM
AEQmit	AEQ-mut	Coe-wt	pLV-AEQmit	mitochondrial matrix	2-100 µM
AEQer	AEQ-mut	Coe-n	pLV-AEQer	ER lumen	10-1000 µM

Supplementary Table 2. Analysis of the statistical significance (p-value, Student's t-test) of data - reported in Fig. 2, panels B-D - for the comparison between different treatments within each PrP genotype. n.s., not significant; n.d., not done.

p-Src/Fyn (Fig. 2B)

**PrP-Tg**	veh	PP3	PP2	sara
veh	-	n.s.	0.05	10^{-8}
	PP3	-	0.005	n.d.
		PP2	-	0.01

**PrP-KO**	veh	PP3	PP2	sara
veh	-	n.s.	0.0005	10^{-6}
	PP3	-	0.005	n.d.
		PP2	-	n.s.

Total p-Tyr (Fig. 2C)

**PrP-Tg**	veh	PP3	PP2	sara
veh	-	n.s.	10^{-5}	10^{-6}
	PP3	-	10^{-4}	n.d.
		PP2	-	n.s.

**PrP-KO**	veh	PP3	PP2	sara
veh	-	n.s.	10^{-6}	10^{-4}
	PP3	-	10^{-5}	n.d.
		PP2	-	n.s.

Peak $[Ca^{2+}]_{pm}$ (Fig. 2D)

**PrP-Tg**	veh	PP3	PP2	sara
veh	-	n.s.	0.05	0.05
	PP3	-	0.05	n.d.
		PP2	-	n.s.

**PrP-KO**	veh	PP3	PP2	sara
veh	-	n.s.	0.01	0.05
	PP3	-	0.01	n.d.
		PP2	-	n.s.
